# Supplementary material for: Network-based prediction approach for cancer-specific driver missense mutations using a graph neural network
Source: BMC Bioinformatics. 2023 Oct 10;24:383. doi: 10.1186/s12859-023-05507-6 (PMC10565986; doi:10.1186/s12859-023-05507-6)
Supplement: Supplementary file 1 — Additional file 1: Figure S1 The mean of ROC-AUC for each classifier when the graph node vector was 16 dimensions. Figure S2 The mean of ROC-AUC for each classifier when the graph node vector was 32 dimensions. Figure S3. The top 30 contributed features in predicting Kim et al. dataset. The top 30 most contributed features are calculated by the average of the absolute SHAP values for each feature. Features prefixed with “graph_feature” are graph node features, with each number corresponding to a dimension of the node feature vectors. The remaining features, such as p-value_w5 and MGAEntropy, represent variant features. (A) The contributed features when using “Cancer pathway + Molecular interaction.” (B) The contributed features when using the initial graph features. [file 12859_2023_5507_MOESM1_ESM.docx]

**Supplementary Figures**


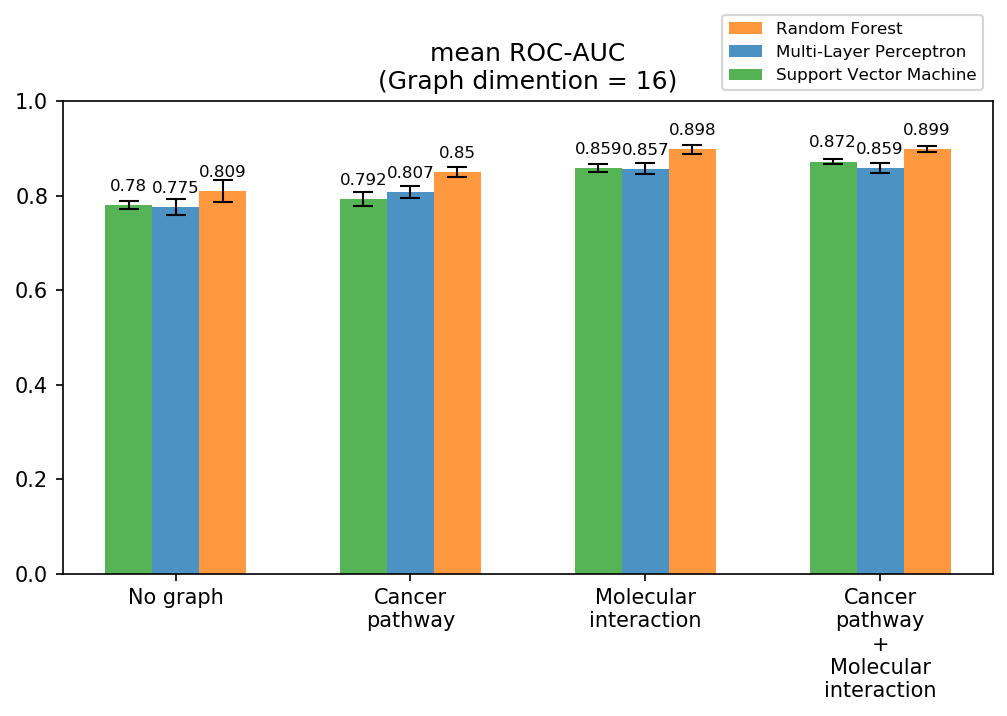


**S Figure 1. The mean of ROC-AUC for each classifier when the graph node vector was 16 dimensions.**


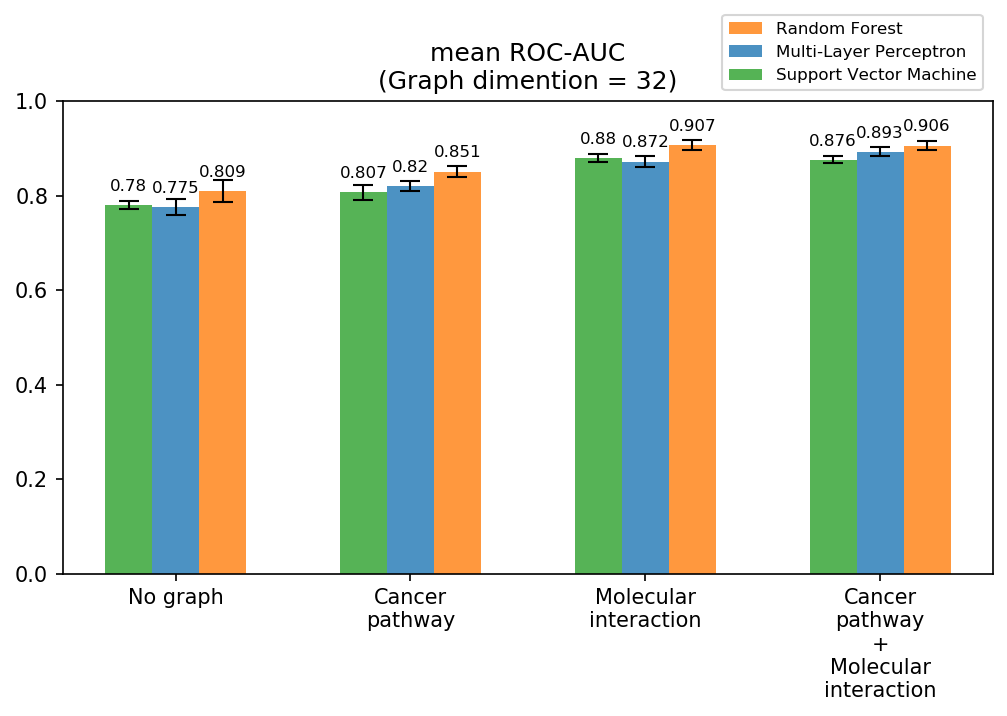


**S Figure 2. The mean of ROC-AUC for each classifier when the graph node vector was 32 dimensions.**


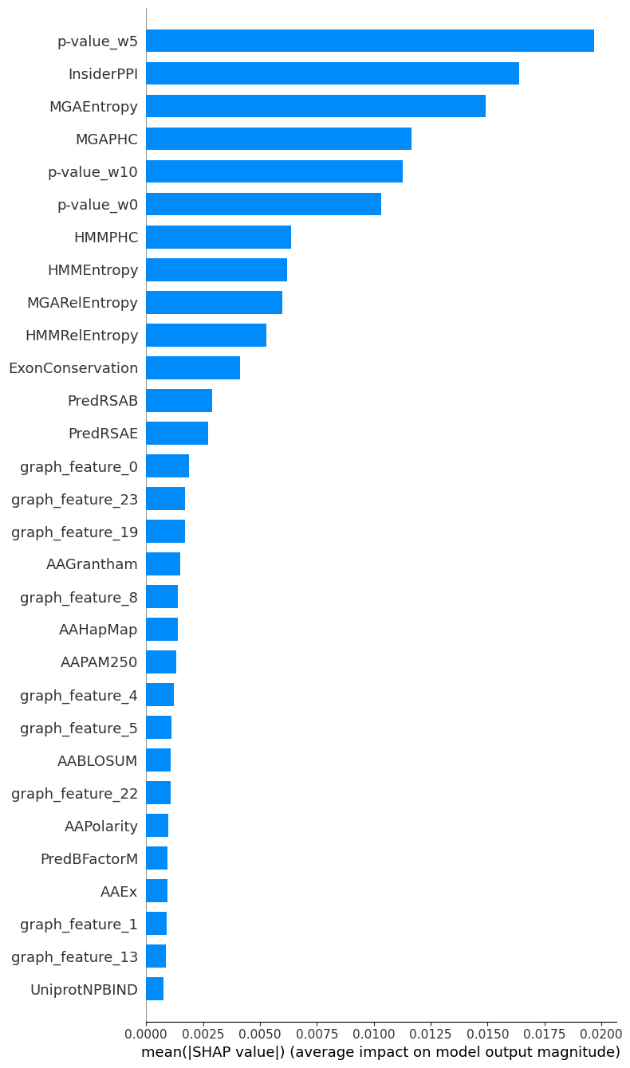

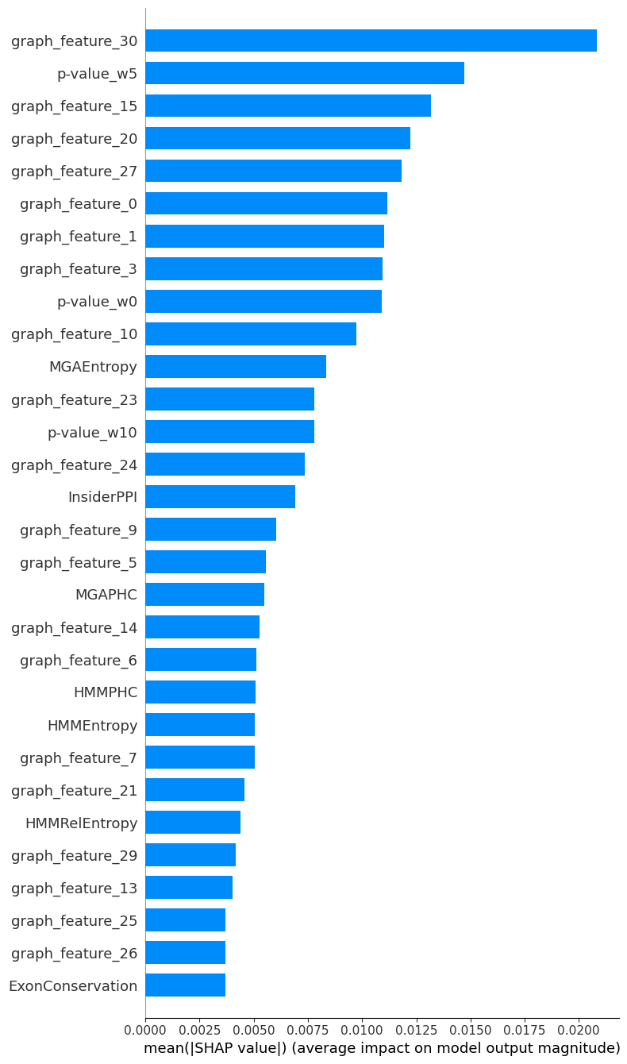


(B)

(A)

**S Figure 3. The top 30 contributed features in predicting Kim et al. dataset.** The top 30 most contributed features are calculated by the average of the absolute SHAP values for each feature. Features prefixed with “graph_feature” are graph node features, with each number corresponding to a dimension of the node feature vectors. The remaining features, such as p-value_w5 and MGAEntropy, represent variant features. (A) The contributed features when using “Cancer pathway + Molecular interaction.” (B) The contributed features when using the initial graph features.
