# Supplementary material for: Network-based prediction approach for cancer-specific driver missense mutations using a graph neural network
Source: BMC Bioinformatics. 2023 Oct 10;24:383. doi: 10.1186/s12859-023-05507-6 (PMC10565986; doi:10.1186/s12859-023-05507-6)
Supplement: Supplementary file 5 — Additional file 5. Supplementary Method. 88 features from SNVBox. [file 12859_2023_5507_MOESM5_ESM.docx]

**Supplementary Method.** The 88 features from SNVBox are below.

AABLOSUM

AACharge

AACOSMIC

AACOSMICvsHapMap

AACOSMICvsSWISSPROT

AAEx

AAGrantham

AAHapMap

AAHGMD2003

AAHydrophobicity

AAMJ

AAPAM250

AAPolarity

AATransition

AATripletFirstDiffProb

AATripletFirstProbMut

AATripletFirstProbWild

AATripletSecondDiffProb

AATripletSecondProbMut

AATripletSecondProbWild

AATripletThirdDiffProb

AATripletThirdProbMut

AATripletThirdProbWild

AAVB

AAVolume

ExonConservation

ExonHapMapSnpDensity

ExonSnpDensity

HMMEntropy

HMMPHC

HMMRelEntropy

MGAEntropy

MGAPHC

MGARelEntropy

PredBFactorF

PredBFactorM

PredBFactorS

PredRSAB

PredRSAE

PredRSAI

PredSSC

PredSSE

PredSSH

PredStabilityH

PredStabilityL

PredStabilityM

RegCompC

RegCompDE

RegCompEntropy

RegCompG

RegCompH

RegCompILVM

RegCompKR

RegCompNormEntropy

RegCompP

RegCompQ

RegCompWYF

UniprotACTSITE

UniprotBINDING

UniprotCABIND

UniprotCARBOHYD

UniprotCOMPBIAS

UniprotDISULFID

UniprotDNABIND

UniprotDOM_Chrom

UniprotDOM_LOC

UniprotDOM_MMBRBD

UniprotDOM_PostModEnz

UniprotDOM_PostModRec

UniprotDOM_PPI

UniprotDOM_RNABD

UniprotDOM_TF

UniprotLIPID

UniprotMETAL

UniprotMODRES

UniprotMOTIF

UniprotNPBIND

UniprotPROPEP

UniprotREGIONS

UniprotREP

UniprotSECYS

UniprotSIGNAL

UniprotSITE

UniprotTRANSMEM

UniprotZNFINGER

InsiderPPI

NumAlignedSpecies

UniprotDensity
